# Promoting veterinary medication safety – Exploring the competencies of community pharmacy professionals in veterinary pharmacotherapy

**DOI:** 10.1016/j.vas.2023.100310

**Published:** 2023-08-19

**Authors:** H. Immonen, M.R. Raekallio, A-R. Holmström

**Affiliations:** a Faculty of Pharmacy, Division of pharmacology and pharmacotherapy, University of Helsinki, Viikinkaari 5 E, 00014, Finland; b Faculty of Veterinary Medicine, Department of Equine and Small Animal Medicine, University of Helsinki, Koetilantie 2, 00014, Finland

**Keywords:** Competence, Medication safety, Pharmacy, Survey, Veterinary pharmacotherapy, Inter-professional

## Abstract

The science of veterinary medicine is currently lacking studies on medication safety, although its importance in protecting animals from medication errors is central. Pharmacy professionals have an important role in ensuring medication safety of both prescription and over-the-counter medications of animals. However, this requires adequate competencies of pharmacy professionals in veterinary pharmacotherapy. The present study aimed to explore the competencies of pharmaceutical staff in community pharmacies in veterinary pharmacotherapy, which factors influence these competencies and what kind of information sources they typically use on veterinary pharmacotherapy.

The study was conducted as a cross-sectional online survey targeted to pharmacy professionals in the Finnish community pharmacies, providing 596 responses. Less than half of the respondents (41%, *n* = 246) are considered to possess good competencies in veterinary pharmacotherapy. A third of the respondents (35%, *n* = 211) would dispense an anti-inflammatory drug for an animal off-label, whereas 24% (*n* = 145) would not interview the pet owner to discover the need for internal parasite medication before dispensing the drug. A small proportion (<1%, *n* = 5) would have dispensed a broad-spectrum internal parasite medication. Approximately a quarter of the respondents (27%, *n* = 159) stated that they acquired information on pharmacotherapy only from the material produced by the manufacturers of veterinary drugs.

The competencies of pharmacy professionals in veterinary pharmacotherapy need to be strengthened in many areas to better promote veterinary medication safety. It should also be ensured that pharmacy professionals can access and use independent, high-quality information on veterinary pharmacotherapy.

## Highlights

• Medication safety and preventive medication risk management in veterinary medicine has been largely unstudied.
• Ensuring medication safety is a multi-professional effort involving veterinarians and other professionals, such as pharmacists.
• The competencies of pharmacy professionals in veterinary pharmacotherapy need to be strengthened in many areas to better promote veterinary medication safety.
• It should be ensured that pharmacy professionals can access and use independent, high-quality information on veterinary pharmacotherapy.

## 1 Introduction

Medication safety is a central part of patient safety in human and veterinary medicine (Donaldson et al., 2017; Oxtoby & Mossop, 2019; Oxtoby et al., 2015). In human medicine, medication safety refers to freedom from unnecessary or potential harm associated with medication use and management process of a patient (i.e., prescribing, storing, dispensing, preparing, and administering the medication and monitoring effects of the treatment) (World Health Organization, 2009). Tackling medication errors (e.g., a veterinarian accidentally administering an oral medication *via* iv-route) requires implementation of a systems approach, stating that errors should be managed proactively by improving the care processes rather than by blaming and shaming individual professionals for committing errors (Reason, 2000; Van Beuzekom et al., 2010).

To date, the field of medication safety and preventive medication risk management in veterinary medicine has been largely unstudied (Oxtoby & Mossop, 2019). In human medicine, the recent two decades have represented a global awakening of healthcare systems and the scientific community to the reality of medication errors (Dzau & Shine, 2020; Panagioti et al., 2019). The prevalence of errors ranges from 0.38% to 1.13% of human hospital admissions resulting in a preventable lethal event (Classen et al., 2010; Landrigan et al., 2010; Makary & Daniel, 2016). Consequently, there is a need to improve medication processes before patients get harmed (Jha et al., 2010). While such evidence in veterinary medicine is lacking, a few existing studies emphasise the role of adequate competencies of professionals and animal caregivers as a central defence against medication errors (Bragg et al., 2010; Oxtoby et al., 2015).

Ensuring medication safety is often a multi-professional effort involving veterinarians and other professionals, such as veterinary nurses and pharmacists (Kinnison et al., 2015; Wells et al., 2014). A substantial part of veterinary medications is dispensed from community pharmacies, whose role is thus essential in securing the medication safety of animals (Fredrickson et al., 2020). Particularly, when dispensing over-the-counter (OTC) medications, a pharmacist may often be the only professional who has contact with the animal owner and can promote the safe and rational implementation of the medication treatment. Consequently, the competencies of the community pharmacists in veterinary pharmacotherapy are pivotal in ensuring the overall medication safety of animals (Fredrickson et al., 2020). While no previous studies exist in this area, the present study aimed to explore the competencies of pharmaceutical staff in community pharmacies in veterinary pharmacotherapy. The specific objectives were to investigate the perceptions of pharmacy professionals of their own competencies with factors influencing their competencies, and to determine the sources used to access information on veterinary pharmacotherapy. Optimization of veterinary medication safety also comprises a central aspect in promoting one-health.

## 2 Material and methods

The present study was conducted as a cross-sectional online survey targeted at pharmacy professionals in Finnish community pharmacies. Survey was selected as a research method as it allows for a variety of methods to recruit participants, collect data and utilize various methods of instrumentation (Ponto, 2015). Survey methodology is often used to describe and explore human behaviour, and it was therefore suitable for the present study exploring the phenomenon of medication safety.

Professionals with Bachelor's or Master's of Pharmacy Degree from any region in Finland could participate the in the study, while students, and pharmacy professionals working outside community pharmacies, were excluded. The material was collected with an electronic questionnaire. A link to the questionnaire and its cover letter was sent twice as a part of an electronic newsletter of the Finnish Pharmacists’ Association in November-December 2020. The newsletter recipients were Finnish Pharmacists’ Association members, representing Finnish pharmacists working in community and hospital pharmacies, industry, medication retail, government or academic institutions. To increase the number of respondents to the survey, a link to the questionnaire was also provided through internal member websites of the Association of Finnish Pharmacies in January 2021. Consequently, the pharmaceutical staff of the Association of Finnish Pharmacies member pharmacies could answer the questionnaire. The survey closed on 31 January 2021. The responses were handled anonymously, and the individual respondents cannot be identified from the results of this study.

The pilot testing of the questionnaire was conducted as an internal pilot where the responding individuals represented the target population of the study. The individuals participating the pilot questionnaire comprised a small convenience sample of community pharmacists (*n* = 6), of which 3 individuals were advocated as a person responsible for veterinary pharmacotherapy in their pharmacy, and 3 were pharmacy professionals working in a community pharmacy, but not being responsible for veterinary pharmacotherapy. As a result of the pilot, minor changes were made to the questionnaire, mainly in terms of the comprehensibility of the questions. The final questionnaire contained structured questions (*n* = 22) and one open-ended question (Appendix 1). The types of structured questions were: single-answer with multiple-choice questions (*n* = 11), multiple-answer questions in which the respondent was able to select more than one answer (*n* = 6), and Likert-scale questions from which the respondent was able to select only one answer (*n* = 5). Some of the structured questions (*n* = 4) also included an “other” - option to which the respondent could give an open answer.

The questionnaire comprised four sections; the first and second sections concerned background information and the self-experienced competency of the respondent in veterinary pharmacotherapy (Table 1). The third section investigated the medication information needs of the respondents and the information sources typically used. The fourth section of the questionnaire presented two theoretical case scenarios with multiple-answer choices to investigate the respondent's level of veterinary pharmacotherapy-related knowledge. The scenarios concerned (i) the purchase of a human analgesic for a dog; and (ii) dispensing an antiparasitic drug for a dog and a cat (Table 1, Appendix 1).

**Table 1:** The structure and questions (n = 23) of the survey. The results of the questions marked with an asterisk are reported in the present article. With regards to the question 18, the article reports only those responses where the option “about the animal legislation” was selected (please see the Appendix 1 for the response options).

Part 1: Background information		Part 2: Competence	Part 3: Perceived needs for information		Part 4: Cases
1. Are there companion animals in your household at the moment or have there ever been? *	2. Are you interested in veterinary pharmacotherapy? *	11. How do you currently perceive your competence in veterinary pharmacotherapy? *	15. Do you think that there is enough information available on veterinary medicines and products when needed?	16. Where do you primarily seek information on veterinary medicines? (Internet vs. printed material)	22. It is a Saturday night, and the customer comes to the pharmacy. He wants to buy an NSAID for his dog who suffers from irregular limping which has now worsened again. He is going to take the dog to a veterinarian on Monday but would like to buy some medicine for the first aid. How do you handle the situation? *
3. Are you a person responsible for veterinary pharmacotherapy at your pharmacy? *	4. Is there a person responsible for veterinary pharmacotherapy at your pharmacy? *	12. How do you perceive providing counselling on veterinary medicines and products? *	17. From what sources do you usually seek information on veterinary medicines and products? *	18. About which subjects would you need more information concerning animal diseases and pharmacotherapy? *	23. The customer has two adult cats (about 4 kg / cat) and one adult dog (25 kg). He has come to the pharmacy to buy some antiparasitic drugs for his pets. How do you solve medical needs of the pets of the customer? *
5. Do you have a Bachelor's or a Master's degree in pharmacy? *	6. In what year did you graduate? *	13. Does a possible lack of information affect your work at the moment?	19. In your opinion, was there/is there enough education on veterinary pharmacotherapy in pharmacy studies?	20. How the studies should be developed?	
7. Have you received any additional training in veterinary medicines and products? *	8. What size is the pharmacy you work in? *	14. How do you update your competence on veterinary pharmacotherapy? *	21. What kind of teaching about veterinary pharmacotherapy would you hope to include to basic education in pharmacy to support work life?		
9. In what area is your workplace located? *	10. How often do you dispense animal prescription or over-the-counter medicines/products? *				

This article reports the results of the structured questions (*n* = 17) focusing on exploring the veterinary pharmacotherapy-related competencies and factors influencing the level of competence of the pharmaceutical staff in community pharmacies, as well as sources of information on veterinary pharmacotherapy (Table 1, Appendix 1). In the context of this study, competence was defined as the “condition of being capable” or “ability” and having “a specific range of skills, knowledge and ability” to provide counselling on veterinary medicines and pharmacotherapy (WHO, 2015; Woit et al., 2020). The data were analysed with SPSS statistical software (IBM SPSS for Windows, version 27.0). The data from five-level Likert-scale questions were combined into three categories for the analysis as the number of answers with extreme values was small compared to the data size. The data were cross-tabulated and subjected to descriptive statistical tests (Mann-Whitney U, Fisher's Exact and Spearman correlation tests). A p-value ≤ 0.05 was considered statistically significant. According to a sample size estimation, a minimum of 369 respondents were needed to have a confidence level of 95% that real value would be within ±5% of the surveyed value, assuming a population proportion of 50% (population size of approximately 8519 pharmacy professionals working in Finnish community pharmacies).

Ethical approval by an Ethics Committee was not acquired as the study was considered a non-medical study that would not require an ethical pre-assessment (Finnish National Board on Research Integrity TENK, 2019). Also, the respondents' personal data or identification information were not handled in the study.

## 3 Results

A total of 596 pharmacy professionals responded to the survey (Bachelor of Pharmacy, *n* = 468 and Master of Pharmacy, *n* = 127). The background information on the respondents is presented in Table 2. The answers were received from all Finnish provinces except Aland; most respondents represented the capital region. The highest number of responses (53%, 312/592) came from medium-sized pharmacies (annual prescription flow from 40,000 to 100,000), followed by answers from large pharmacies (34%, 203/592, annual prescription flow over 100,000) and small pharmacies (13%, 77/592, annual prescription flow less than 40,000). The majority of respondents (88%, 522/592) expressed their interest in veterinary pharmacotherapy. Approximately 72% (428/595) reported that their pharmacy had a pharmacy professional advocated as a person responsible for veterinary pharmacotherapy.

**Table 2:** Respondents’ Background information (*n* = 595).

Background information	Number of respondents (n)*	% (n)
**Education** • Bachelor of Science in Pharmacy • Master of Science in Pharmacy	468 (595) 127 (595)	79 (468) 21 (127)
**Year of graduation** • 1999 or before • 2000–2009 • 2010–2019	198 (595) 149 (595) 248 (595)	33 (198) 25 (149) 42 (248)
**A companion animal owner** • Yes • No	502 (597) 95 (597)	84 (502) 16 (95)
**Interested in veterinary medicine** • Yes • No	522 (592) 70 (592)	88 (522) 12 (70)
**Respondent is a person responsible for veterinary pharmacotherapy** • Yes • No	123 (595) 472 (595)	21 (123) 79 (472)
**There is a person responsible for veterinary pharmacotherapy at the respondent's pharmacy** • Yes • No	428 (595) 167 (595)	72 (428) 28 (167)
**Annual number of dispensed prescriptions at the respondent's pharmacy** • less than 40 000 • 41 000 – 100 000 • over 100 000	77 (592) 312 (592) 203 (592)	13 (77) 53 (312) 34 (203)

⁎ Not all respondents answered all the questions of the questionnaire.

### 3.1 Perceptions of pharmacy professionals on their competencies in veterinary pharmacotherapy

Approximately 41% of the respondents (246/595) considered their competencies in medication treatment of animals to be moderately good or very good. In comparison, 21% of the respondents (125/595) considered their competencies very poor or moderately poor. The remaining respondents (38%, 224/595) rated their knowledge as neither good nor poor. Of the respondents, 21% (123/595) were advocated as a person responsible for veterinary pharmacotherapy in their pharmacy. Approximately 86% (106/123) of the pharmacy professionals responsible for pharmacotherapy perceived their skills as moderately good or very good. They also felt that their competencies were better than those respondents (79%, 472/595) who were not responsible for veterinary pharmacotherapy (p ˂ 0.001). Respondents who rated their knowledge as very or moderately poor often found it more difficult to provide counselling on veterinary medicines than those who rated their knowledge as moderately good or very good (p ˂ 0.001).

### 3.2 Competence in veterinary pharmacotherapy in practice situations

Approximately 35% of the respondents (211/595) indicated their readiness to dispense ketoprofen, and one respondent a paracetamol product, to a dog when the respondents were asked to respond to a hypothetical veterinary patient case (Table 1; Appendix 1). Of these respondents, 65% (138/212) would check the dose from the pharmacy's own veterinary medicine information folder, whereas 26% (56/212) would check the dose from the Internet and 8% (18/212) from both of these information sources. The respondent's experience of own competence in veterinary pharmacotherapy did not appear to affect whether one was ready to dispense the drug. However, a proportion of respondents (14%, 84/596) perceived that they needed more information on veterinary legislation which regulates their professional actions in such situations. In the other veterinary patient case, 24% of respondents (145/595) would not have ensured the actual need for medication treatment by any means before dispensing the parasitic drug (Table 1; Appendix 1), whereas a small proportion (<1%, 5/595) would have dispensed a broad-spectrum drug. The majority of these respondents (74%, 108/145) had rated their own competence in veterinary pharmacotherapy as poor or neither good nor bad. Overall, the respondents who were advocated as a person responsible for veterinary pharmacotherapy in their pharmacies were more likely to provide answers supporting the safe and rational use of medicines in the parasite case (*p* = 0.002) compared to other respondents. However, in the pain management case, the difference between these groups was non-significant.

### 3.3 Factors influencing the competencies in veterinary pharmacotherapy

**Fig 1:**
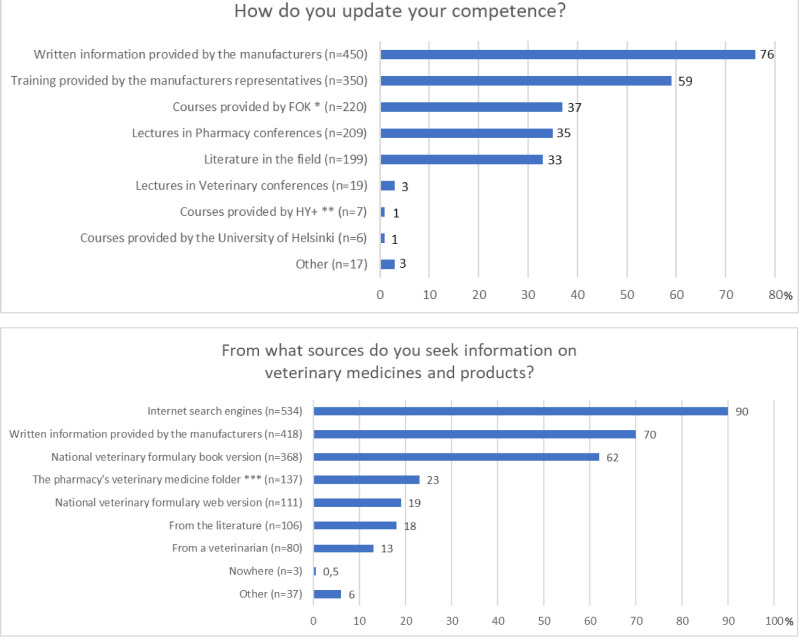
The means for updating the competence and sources of information used on veterinary medicines and products by the respondents (*n* = 596). The respondents were able to select all the applicable answer options. * The Pharmaceutical Learning Centre ** University of Helsinki Centre for Continuing Education *** A folder comprising central information on the veterinary medications maintained by the pharmacies.

Approximately 84% of the respondents (502/596) reported themselves as current or previous companion animal owner, whereas 16% (95/596) had never owned a companion animal. The animal owners perceived their competence in veterinary pharmacotherapy better when compared to those who had never owned an animal (p ˂ 0.001). Respondents who dispensed veterinary drugs and products daily or weekly also perceived their competence better than those who delivered these products less frequently (p ˂ 0.001). The majority of respondents (75%, 448/596) occasionally participated in veterinary pharmacotherapy training, whereas 16% (94/596) did not attend such educational activities at all. Those respondents who reported attending training frequently perceived their competencies better than those who reported attending training less often (p ˂0.001). A quarter of the respondents (27%, 59/595) stated that to acquire their knowledge only from the sources provided by the manufacturers of veterinary medicines and products. Those respondents (73%, 436/595) who used information sources other than those provided by manufacturers rated their own competence better than those who did not use other information sources (p ˂ 0.001). However, 90% of respondents (534/596) searched for information on veterinary medicines and products from Internet search engines (Fig. 1). The next most applied information sources were the written material provided by the manufacturers (brochures and training) and the Pharmaca Fennica Veterinaria printed version. In general, those who had graduated before 1994 and those who graduated after 2010 felt that they had better competencies in veterinary pharmacotherapy than those who had graduated between 1995 and 2009. However, there was a negative correlation between the year of graduation and the competencies experienced by the respondents (ρ = - 0,13; *p* = 0.002). The factors influencing the competencies are summarized in the Table 3.

**Table 3:** A summary of the identified factors leading to better perceived competencies in veterinary pharmacotherapy by the responding community pharmacy professionals (*n* = 595).

Factor
Being a pharmacy professional responsible of veterinary pharmacotherapy in one's own pharmacy
Owning a companion animal
Dispensing veterinary drugs/products daily or weekly
Frequent participation in training on veterinary pharmacotherapy
Using other information sources on veterinary pharmacotherapy than the ones provided by pharmaceutical manufacturers
Graduation before the year 1994
Graduation after the year 2010

## 4 Discussion

The present study indicates a wide variation between community pharmacists in their competencies in veterinary pharmacotherapy. The majority of the respondents experienced gaps in their current level of knowledge and skills, and only less than half felt content with their competencies. The community pharmacists’ perception of their knowledge also affected how the counselling on veterinary pharmacotherapy was experienced. Concerning preventive risk management in veterinary medication safety, these findings are pivotal, as the experienced discomfort may lead to the omission of medication counselling, which has been identified as a central contributing factor to medication errors in human medicine (Showande & Laniyan, 2022; Walker et al., 2014).

The majority of the respondents’ pharmacies had advocated a pharmacy professional responsible for veterinary pharmacotherapy and who, in this study, were also found to possess the best experience of their competencies in veterinary pharmacotherapy. This distinctive system may, at least to some extent, support the safe and rational use of veterinary medicines and may represent one factor safeguarding veterinary medication safety in the current Finnish veterinary care system. While the current academic literature does not identify such positions in other countries, establishing persons responsible for veterinary pharmacotherapy in community pharmacies could be internationally promoted to support a veterinary patient's care path from the clinic through the pharmacy to home. Moreover, such positions could be promoted by developing international standards for the competencies of pharmacy personnel responsible for veterinary pharmacotherapy in their pharmacies. This would also enable better utilisation of community pharmacies in supporting veterinarians in preventive medication risk management of their animal patients and create coordinated collaboration with veterinary clinics and pharmacies. However, this study indicates that education should be strengthened not only for these advocated professionals, but especially for those community pharmacists whose profile does not comprise any specific responsibilities related to veterinary pharmacotherapy, as the training activity correlated with the level of perceived competence. A few other studies represent similar findings by emphasising the need for more education for pharmacy staff in veterinary pharmacotherapy (Ceresia et al., 2009; Frankel et al., 2016; Fredrickson et al., 2020; Theberge & Sehgal, 2016). Consequently, the detected deficiencies in the competencies of pharmacy professionals may represent a key development area internationally.

Our study found that in Finnish community pharmacies, the information on veterinary pharmacotherapy was largely obtained from materials provided by pharmaceutical manufacturers. This finding is somewhat worrying, as to optimally support the safe and rational use of medicines, the information provided to medicine users should be based on an independent source (Mononen et al., 2018; Prusti et al., 2012). In addition, if the information is retrieved from the Internet, representing the commonest source or information in the present study, it should be ensured that the professionals can assess the source reliability (Mononen et al., 2018; Närhi et al., 2008; Prusti et al., 2012). Consequently, more research is warranted to explore the root causes of pharmacy professionals not primarily using independent, evidence-based information sources in medication counselling and whether such easy-to-access and easy–to–use information sources are lacking and should be developed as an inter-professional action between veterinarians and pharmacists.

The present study also investigated the competencies of pharmacy professionals in two separate OTC areas. The first was the off-label use of medicines as a primary risk factor affecting medication safety (Fitzgerald et al., 2006). The development of antiparasitic resistance is another important risk factor for animal and human medication safety, which could be alleviated by adequate medication counselling in community pharmacies (Kaplan & Vidyashankar, 2012; Rousseau et al., 2022). Our results suggest that the awareness of pharmacy professionals about their authority concerning the off-label use of medicines should be improved. Approximately one-third of the respondents would have sold an NSAID licensed for human use, knowing it would be used for an animal. However, only a veterinarian is allowed to decide to use a medicine under the cascade, and only if there is no authorised veterinary medicinal product for that condition (Regulation, 2019/6). A similar lack of knowledge about legislation concerning dispensing of veterinary drugs has been reported in other countries amongst pharmacy professionals (Monie et al., 2006). The results of our study also revealed the need to increase the awareness of pharmacy professionals about the policies and their role in reducing antiparasitic resistance. One-fourth of the respondents would have sold antiparasitic drugs for small animal use without interviewing the owner, and some would even have sold a wide-spectrum medicine. However, the indication for its use did not exist. However, to reduce the development of resistance, routine antiparasitic treatment is not recommended for low-risk animals in areas with a low prevalence of parasite infections (European Scientific Counsel Companion Animal Paracites (ESCCAP), 2021; Pullola et al., 2006). Unnecessary use of wide-spectrum drugs should also be avoided, and to target the treatments for the diagnosed or suspected parasites (Castro et al., 2019; Thompson & Roberts, 2001).

The survey respondents were primarily interested in veterinary pharmacotherapy; most were current or former animal owners. This represents a potential limitation to our study and may bias the results, although there was also a considerable number of respondents with no experience on owning an animal. The competence assessment was based on the respondent's experience and two imaginary example cases. Although perceptions of pharmacy professionals on their own competence can be considered one measure of competence (Ginsburg et al., 2013; Kallio et al., 2021), unambiguous conclusions about the actual level of competence in practical work can only be drawn with additional evidence. The content validity of the survey instrument may have also been affected by the small convenience sample of the pilot respondents. In the future, it would be necessary to further investigate the role of pharmacy professionals and the possibilities of influencing the medication safety of animals.

## 5 Conclusions

Community pharmacists represent an important healthcare professional group supporting veterinarians in preventive medication safety risk management of animals. However, pharmacy professionals' competencies in veterinary pharmacotherapy must be strengthened in many areas, especially in high-risk situations such as off-label use of medicines and antiparasitic resistance. These findings call for educational actions in pharmacy academia and community pharmacies to reduce possible fear and discomfort associated with medication counselling on veterinary pharmacotherapy. It should also be ensured that pharmacy professionals can access and use independent, high-quality information on veterinary pharmacotherapy.

## Funding

Open access funded by Helsinki University Library. This research did not receive any other specific grant from funding agencies in the public, commercial, or not-for-profit sectors.

## Ethical statement

Ethical approval by an Ethics Committee was not acquired as the study was considered a non-medical study that would not require an ethical pre-assessment (Finnish National Board on Research Integrity TENK, 2019). Also, the respondents' personal data or identification information were not handled in the study. The study did not involve any animals, and therefore, adherence to ARRIVE guidelines (Animal Research: Reporting of In Vivo Experiments) are not reported.

## Declaration of Competing Interest

The authors declare that they have no known competing financial interests or personal relationships that could have appeared to influence the work reported in this paper.

## Appendix Supplementary materials

Image, application 1
