# Supplementary material for: Promoting veterinary medication safety – Exploring the competencies of community pharmacy professionals in veterinary pharmacotherapy
Source: Vet Anim Sci. 2023 Aug 19;21:100310. doi: 10.1016/j.vas.2023.100310 (PMC10468355; doi:10.1016/j.vas.2023.100310)
Supplement: Supplementary file 1 [file mmc1.docx]

**Appendix 1:** The study questionnaire

**Part 1: Background information**

1. Are there companion animals in your household at the moment or have there been in the past?

- Yes, I have companion animals at the moment.
- No, I don’t have any companion animals at the moment, but I have in the past.
- No, I have never had any companion animals.

2. Are you interested in veterinary pharmacotherapy?

- Yes
- No

3. Are you a person responsible for veterinary pharmacotherapy at your pharmacy?

- Yes
- No

4. Is there a person responsible for veterinary pharmacotherapy at your pharmacy?

- Yes
- No

5. Do you have a bachelor’s or a master’s degree in pharmacy?

- A Bachelor’s
- A Master’s

6. In what year did you graduate?

- 2015-2019
- 2010-2014
- 2005-2009
- 2000-2004
- 1995-1999
- 1990-1994
- Before 1990

7. Have you received any additional training in veterinary medicines and products?

- Not at all.
- Little (I participate to in-service training sometimes).
- A lot (I participate to in-service training regularly).

8. What size is the pharmacy you work in?

- Small (less than 40 000 prescriptions dispensed a year)
- Medium (41 000 - 100 000 prescriptions dispensed a year)
- Large (over 100 000 prescriptions dispensed a year)

9. In what area is your workplace located?

- Uusimaa
- Varsinais-Suomi
- Satakunta
- Häme
- Pirkanmaa
- Päijät-Häme
- Kymenlaakso
- Etelä-Karjala
- Etelä-Savo
- Pohjois-Savo
- Pohjois-Karjala
- Keski-Suomi
- Etelä-Pohjanmaa
- Pohjanmaa
- Keski-Pohjanmaa
- Pohjois-Pohjanmaa
- Kainuu
- Lappi
- Ahvenanmaa

10. How often do you dispense animal prescription or over-the-counter medicines/products?

- Daily
- Weekly
- Monthly
- A few times a year
- Less than once a year
- Never

**Part 2: Competence**

11. How do you currently perceive your competence in veterinary pharmacotherapy?

- Very poor
- Moderately poor
- Neither good nor bad
- Moderately good
- Very good

12. How do you perceive providing councelling on veterinary medicines and products? It is...

- Very hard
- Moderately hard
- Neither hard nor easy
- Moderately easy
- Very easy

13. Does a possible lack of information affect your work at the moment?

- A lot
- Quite a lot
- Neither much nor little
- A little
- Quite a little

14. How do you update your competence on veterinary pharmacotherapy? You can select several options.

- Courses provided by HY+ (a continuing learning center by the University of Helsinki, Finland).
- Courses provided by FOK (a non-profit pharmacy continuing learning company in Helsinki, Finland).
- Lectures in the annual Pharmacy conference in Finland.
- Lectures in the annual Veterinary conference in Finland.
- Literature in the field.
- Information provided by the manufacturers.
- Information provided by the medicine agents by drug companies.
- Courses provided by the University of Helsinki.
- Other, please specify.

**Part 3: Perceived needs for information**

15. Do you feel that there is enough information available on veterinary medicines and products when needed?

- Yes
- No

16. Where do you primarily seek information on veterinary medicines? (Internet vs. printed material)

- From the Internet.
- From printed material.

17. From what sources do you usually seek information on veterinary medicines and products? You can select several options.

- From the literature.
- Pharmaca Fennica Veterinaria (web version).
- Pharmaca Fennica Veterinaria (book version).
- From a veterinarian.
- Manufacturers leaflets/courses.
- The pharmacy’s own veterinary medicine folder.
- Internet search engines (e.g. Google).
- Nowhere.
- Other, please specify.

18. About which subjects would you need more information concerning animal diseases and pharmacotherapy? You can select several options.

- Treating endo- and ectoparasites.
- Pain management.
- Wound care.
- Gastrointestinal problems.
- Skin problems.
- Behaviour problems.
- How to give medicine to an animal.
- Prescription medicines.
- Differences in medical care between different species.
- Legislation regarding animal medicines.
- Other, please specify.

19. In your opinion, was there/is there enough education on veterinary pharmacotherapy in pharmacy studies?

- Yes
- No

20. If you answered yes to the previous question, how the studies should be developed?
(Open answer)

21. What kind of teaching about veterinary pharmacotherapy would you hope to include to basic education in pharmacy to support work life? You can select several options.

- A compulsory course.
- An optional course.
- Teaching requiring attendance in connection with other courses (e.g., lectures/project work).
- More optional teaching in connection with other courses (e.g., lectures/project work).
- A veterinary medicine-related task included in the internship period (e.g., updating the pharmacy’s own veterinary medicine folder).
- Other, please specify.

**Part 4: Cases**

Please answer based on your current own knowledge and do not use any available sources of information, thank you!

22. It is a Saturday night, and the customer comes to the pharmacy. He wants to buy an NSAID for his dog who suffers from irregular limping, which has now worsened again. He is going to take the dog to a veterinarian on Monday but would like to buy some medicine for the first aid. How do you handle the situation? You can select several options.

- I sell the customer ketoprofen and check the dose from the pharmacy’s own veterinary medicine folder.
- I sell the customer ketoprofen and check the dose from the Internet.
- I sell the customer paracetamol and check the dose from the pharmacy’s own veterinary medicine folder.
- I sell the customer paracetamol and check the dose from the Internet.
- I advise the customer to contact the vet.

23. The customer has two adult cats (about 4kg/cat) and one adult dog (25kg). He has come to the pharmacy to buy some antiparasitic drugs for his pets. How do you solve the medical needs of the pets of the customer? You can select several options.

- I ask if the customer wants single doses or multi-day regimen and I choose the right medicines according to the weight.
- I ask if the customer wants the medicines in paste form or in tablets and I choose the right medicines according to the weight.
- I carefully survey the customers pets ages and living habits and I sell suitable medicines according to answers.
- I carefully survey the customers pets ages and living habits, consider if medication is needed and either sell suitable medicines or instruct the owner to contact the vet
- I instruct the owner to contact the vet to determine if the pets have any endoparasites that need to be treated.
- I sell an endoparasite test package to the customer and advise him to act depending on the results.
- I suggest that all pets should be treated with broad-spectrum medication so that all the parasites will die and not move from one pet to another.
- I suggest that all pets should be treated with narrow-spectrum medication in order to avoid resistant endoparasite strains.
